# Telemedicine-Guided Pericardiocentesis for Cardiac Tamponade in Primary Mediastinal Large B-cell Lymphoma

**DOI:** 10.7759/cureus.116854

**Published:** 2026-09-24

**Authors:** Victor A Cab Morales, Edgar A Montemayor Garza, César A Mascorro-Cienfuegos, Jorge L Aguilar-Meza, Nury A de la Cruz de León, Uriel Chavarría Martínez

**Affiliations:** 1 Internal Medicine, Hospital Universitario Dr. José Eleuterio González, Monterrey, MEX; 2 Nephrology, Hospital Universitario Dr. José Eleuterio González, Monterrey, MEX; 3 Cardiology, Hospital Universitario Dr. José Eleuterio González, Monterrey, MEX; 4 Microbiology, Universidad Autónoma de Nuevo León, Monterrey, MEX; 5 Pulmonary and Critical Care Medicine, Hospital Universitario Dr. José Eleuterio González, Monterrey, MEX

**Keywords:** cardiac tamponade, mediastinal mass, non-hodgkin lymphoma, pericardial effusion, pericardiocentesis, primary mediastinal lymphoma, rural health services, telemedicine

## Abstract

Pericardial effusion has infectious, inflammatory, neoplastic, traumatic, cardiac, vascular, and idiopathic causes. When effusion accumulates rapidly enough to compromise ventricular filling, cardiac tamponade develops and becomes a life-threatening emergency requiring urgent drainage, a procedure conventionally performed by a cardiologist or cardiothoracic surgeon and rarely available on site at rural or resource-limited hospitals. Malignant pericardial effusion is most often attributed to lung and breast carcinoma, while lymphoma is the leading hematologic cause. Tamponade as the presenting feature of non-Hodgkin lymphoma, however, is distinctly uncommon.

We describe a 40-year-old man from a rural community in Nuevo León, Mexico, with three weeks of intermittent chest tightness, diaphoresis, unquantified fever, and approximately 7 kg of unintentional weight loss, followed by progressive exertional dyspnea, orthopnea, and paroxysmal nocturnal dyspnea. He presented to a rural hospital with jugular venous distension, muffled heart sounds, and lower-limb edema, without pulsus paradoxus. Echocardiography demonstrated a severe pericardial effusion measuring up to 4.6 cm with tamponade physiology. With no cardiologist on site and hemodynamic collapse imminent, an internist performed pericardiocentesis under real-time telemedicine guidance from a cardiologist, draining serosanguineous fluid whose biochemical profile suggested tumor involvement. Contrast-enhanced chest computed tomography at the referral center revealed a 14.4 × 11.8 × 11.3 cm anterior mediastinal mass with necrotic areas. Video-assisted thoracoscopic surgery allowed pericardial window creation and tumor biopsy, which established a CD20-positive primary mediastinal large B-cell lymphoma (PMBCL). Chemotherapy with R-CHOEP (rituximab, cyclophosphamide, doxorubicin, vincristine, etoposide, and prednisone) was initiated, with resolution of hemodynamic instability and no recurrence of tamponade during the reported follow-up.

This case illustrates that malignancy warrants early consideration in unexplained tamponade and that real-time telemedicine guidance may allow a non-specialist physician to undertake a life-saving bedside procedure where specialist care is otherwise unavailable, an approach that merits further evaluation in rural and underserved settings.

## Introduction

The pericardial space normally contains 10-50 mL of fluid; effusion beyond this volume has infectious, inflammatory, neoplastic, traumatic, cardiac, vascular, or idiopathic causes [1,2]. Tamponade develops when the accumulated volume raises intrapericardial pressure enough to compress the cardiac chambers and impair diastolic filling, producing obstructive shock; the rate of accumulation, not the absolute volume, determines whether tamponade occurs [1]. Neoplastic disease accounts for a substantial share of moderate-to-large effusions in referral series, most often lung or breast carcinoma among solid tumors and lymphoma among hematologic malignancies, and carries prognostic weight in patients with cancer [3,4].

Definitive management requires pericardiocentesis or surgical drainage, procedures conventionally performed by a cardiologist, intensivist, or cardiothoracic surgeon. Specialists are often unavailable at rural or resource-limited hospitals. This leaves a hemodynamically unstable patient with two imperfect options: interfacility transfer, which risks decompensation en route, or an emergency bedside procedure without direct specialist support. Telemedicine, real-time audio, video, or image transmission linking a remote specialist to a bedside operator, has increasingly been proposed as a way to close this gap, extending specialist-level guidance for time-sensitive procedures to settings where the specialist cannot be physically present.

Tamponade as the initial manifestation of non-Hodgkin lymphoma is rare, and published experience consists largely of individual case reports concerning mainly Hodgkin lymphoma [4]. Primary mediastinal large B-cell lymphoma (PMBCL) is recognized in the current World Health Organization classification of lymphoid neoplasms as a distinct clinicopathologic entity, arising from thymic B cells and typically presenting as a bulky anterior mediastinal mass in young adults, often with local invasion of contiguous structures such as the pleura, pericardium, and great vessels [5]. We report a patient in whom tamponade was the presenting event of PMBCL, and in whom pericardiocentesis, performed by an internist at a rural hospital under continuous telemedicine supervision by a cardiologist, illustrates how this model can extend emergency specialist-level care to settings where it would otherwise be unavailable, followed by timely referral, tissue diagnosis, and a favorable outcome.

## Case presentation

A 40-year-old man from a rural community in Nuevo León, Mexico, with no known immunosuppression and no history of tuberculosis contact, presented with a three-week history of mild, intermittent, progressive chest tightness, diaphoresis, unquantified fever, and approximately 7 kg of unintentional weight loss over one month. One week before evaluation, he developed progressive exertional dyspnea, orthopnea, and paroxysmal nocturnal dyspnea. A private cardiologist found low QRS voltage on electrocardiography and, on bedside echocardiography, a severe pericardial effusion up to 4.6 cm, prompting referral to the nearest rural hospital.

On admission to the rural hospital, vital signs were as follows: blood pressure 100/70 mmHg, heart rate 109 beats/min, respiratory rate 24 breaths/min, and temperature 36.7°C. Examination showed jugular venous distension to the earlobe, muffled heart sounds, decreased breath sounds at both lung bases, and lower-limb edema; the patient was normotensive, and pulsus paradoxus was notably absent.

Initial laboratory studies at the rural hospital showed hemoglobin 13.2 g/dL, hematocrit 45.5%, leukocytes 10.2 × 10³/µL, neutrophils 8.3 × 10³/µL, and platelets 423 × 10³/µL, with an international normalized ratio of 1.16. Basic chemistry showed glucose 113 mg/dL, creatinine 0.9 mg/dL, blood urea nitrogen 20 mg/dL, uric acid 7.9 mg/dL, albumin 4.1 g/dL, sodium 142 mEq/L, chloride 103 mEq/L, potassium 4.8 mEq/L, and lactate dehydrogenase 475 IU/L. Serologic testing was negative for HIV, hepatitis B, and hepatitis C.

Electrocardiography at the rural hospital confirmed low QRS voltage across the limb leads. Chest radiography showed marked cardiomegaly with a globular cardiac silhouette (Figure 1). Venous blood gas showed pH 7.31, partial pressure of carbon dioxide 27 mmHg, partial pressure of oxygen 36 mmHg, lactate 4.1 mmol/L, and bicarbonate 16.7 mmol/L.

**Figure 1:**
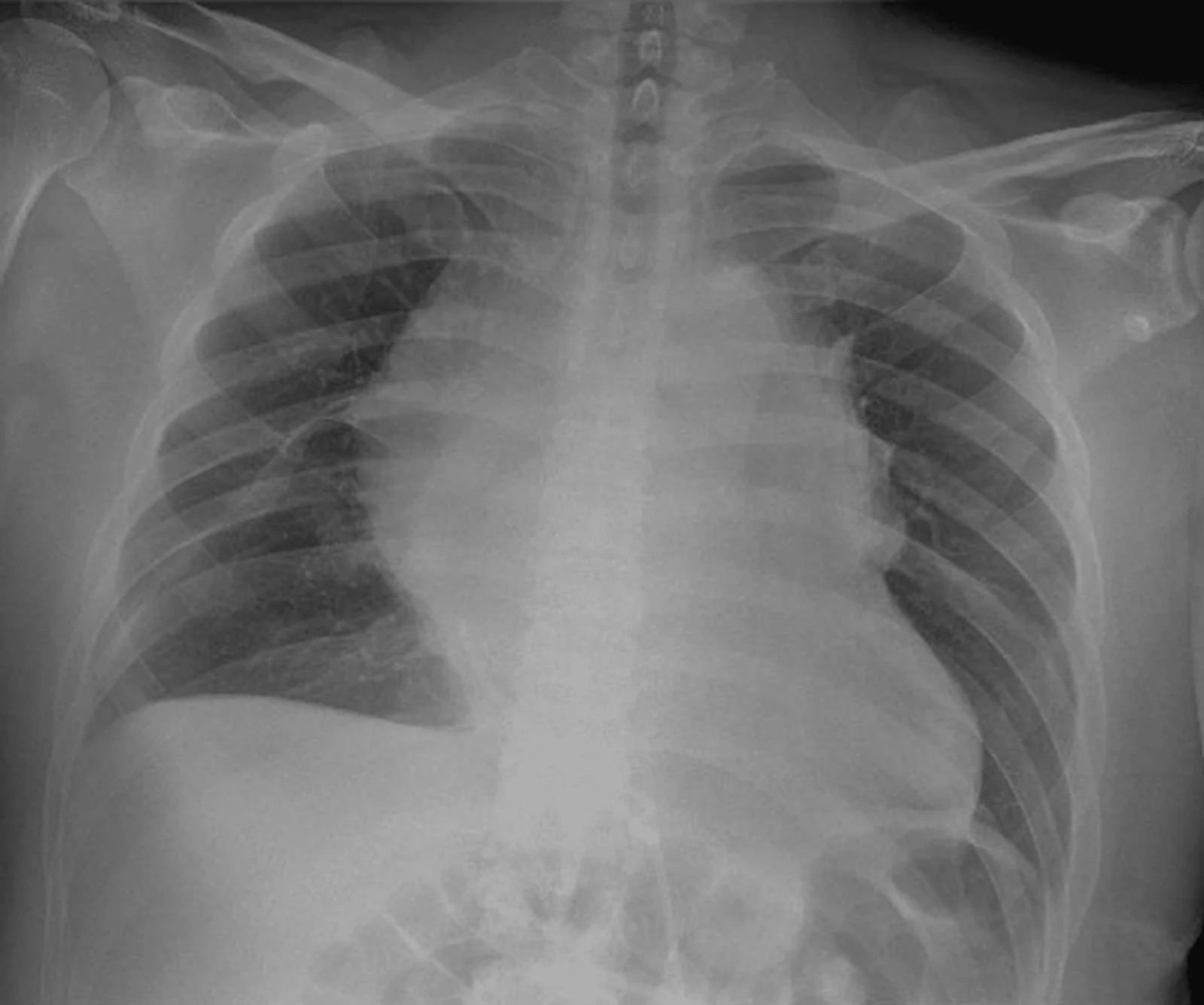
Chest radiograph showing marked cardiomegaly Chest radiograph obtained at the rural hospital, showing marked cardiomegaly with a globular cardiac silhouette, consistent with a large pericardial effusion.

Point-of-care transthoracic echocardiography demonstrated a severe pericardial effusion measuring up to 4.6 cm with tamponade physiology: 33% transmitral and 44% transtricuspid respiratory flow variation, diastolic collapse of the right ventricle and right atrium, dilation of the inferior vena cava to 2.4 cm, and inspiratory interventricular septal shift consistent with ventricular interdependence.

No cardiologist was available on site. Given imminent tamponade and impending hemodynamic collapse, an internist performed ultrasound-guided pericardiocentesis via a subxiphoid approach, under real-time guidance from a cardiologist connected by video call (Figure 2). Reddish, serosanguineous fluid was obtained, and a closed-circuit drainage system was placed, yielding 600 mL over the first 24 hours and 500 mL over the following 24 hours. Interval ultrasound showed reduction of the effusion to 1.8 cm, and contrast-enhanced chest computed tomography was obtained the next day; the patient was transferred to the tertiary referral center on hospital day 3.

**Figure 2:**
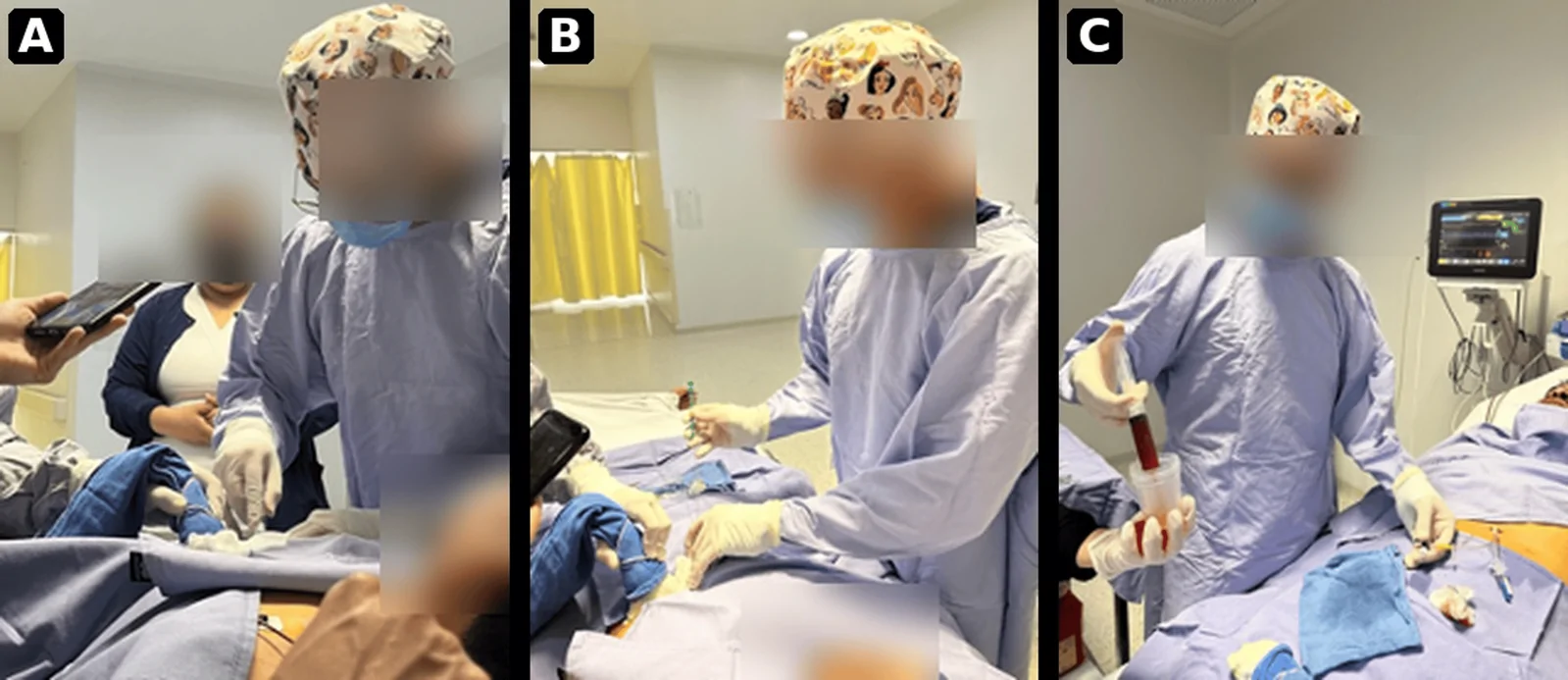
Telemedicine-guided pericardiocentesis Telemedicine-guided pericardiocentesis performed by an internist at the rural hospital. A: a remote cardiologist provides real-time guidance by video call during the procedure; B, C: ultrasound-guided subxiphoid drainage of serosanguineous pericardial fluid. Identifiable features of staff and patient have been obscured.

Pericardial fluid analysis showed pH 8.0, density 1.010, 2,155 cells/µL with 80% lymphocytes and 11% neutrophils, glucose 64 mg/dL, protein >6 g/dL, cholesterol 69 mg/dL, lactate dehydrogenase >2,000 IU/L, and adenosine deaminase 200 IU/L, an exudate compatible with tumor invasion. Aerobic bacterial culture, acid-fast bacilli smear and mycobacterial culture, and potassium hydroxide preparation with fungal culture were all negative.

The patient was promptly referred to the nearest tertiary care center. Contrast-enhanced chest and abdominal computed tomography on admission showed a right pleural effusion of approximately 20% with compressive atelectasis and a residual pericardial effusion of 3.7 cm with the drainage catheter in adequate position. At the anterior mediastinum, a heterogeneous, solid, infiltrative soft-tissue mass measuring 14.4 × 11.8 × 11.3 cm was identified, with multiple internal hypodense areas suggestive of necrosis; the mass abutted the sternal manubrium without evidence of bony erosion and encased the right pulmonary artery and aortic arch across their full circumference, displacing both posteriorly and narrowing their lumen without evidence of thrombosis, with reactive-appearing mediastinal lymph nodes (Figure 3). The initial radiologic impression favored a germ cell tumor, to be correlated with histopathology; alpha-fetoprotein and beta-human chorionic gonadotropin were subsequently found within normal limits, arguing against this.

**Figure 3:**
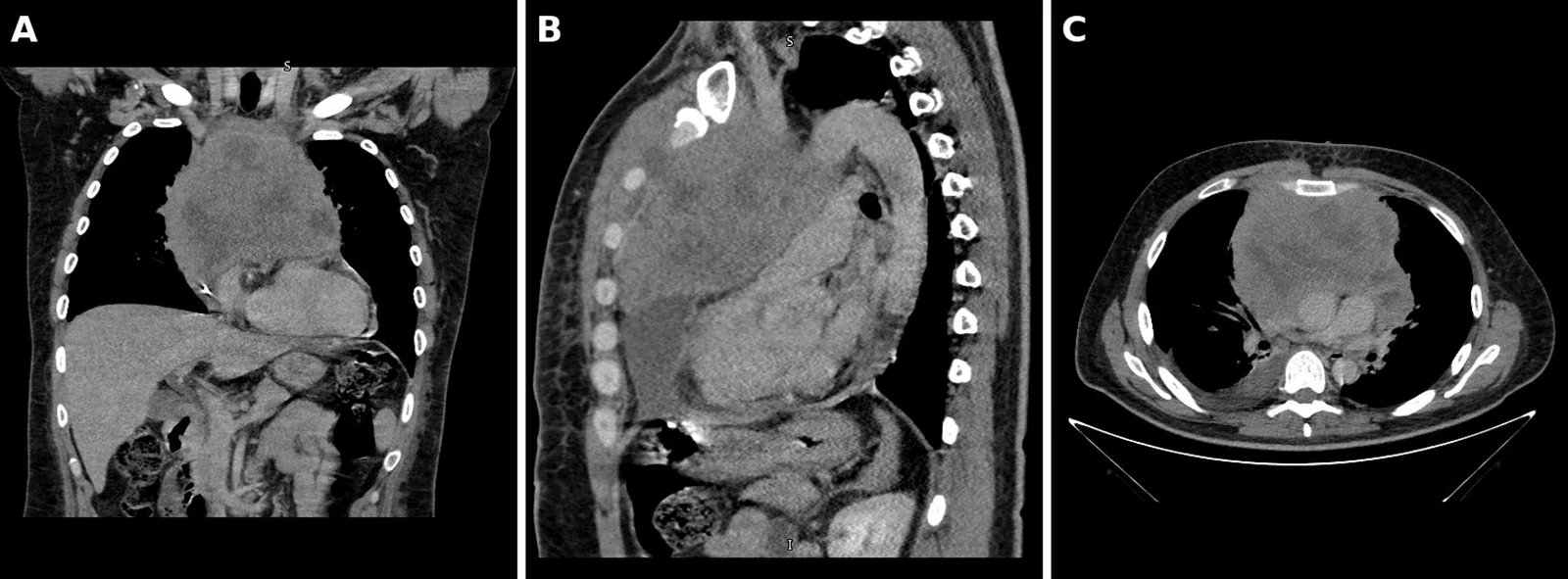
Contrast-enhanced chest CT showing anterior mediastinal mass Contrast-enhanced chest computed tomography showing a large anterior mediastinal mass with internal necrosis, encasing the aortic arch and right pulmonary artery, with associated pericardial and right pleural effusions. A: coronal view; B: sagittal view; C: axial view.

The cardiothoracic surgery service performed video-assisted thoracoscopic surgery for drainage of the pericardial effusion, creation of a pericardial window, and biopsy of the mediastinal mass. Cytology of the pericardial fluid, reported the following day, showed hemorrhagic material with acute inflammatory cells; immunocytochemistry was positive for CD20 and Ki-67 and negative for BCL2 and C-MYC, reported as diffuse large B-cell lymphoma. Before the biopsy result returned, the patient developed right upper-extremity swelling; venous ultrasound demonstrated thrombosis of the right internal jugular vein with partial involvement of the subclavian and axillary veins, managed with enoxaparin during hospitalization, and transitioned to apixaban at discharge.

Initial histopathology from the thoracoscopic biopsy reported a large B-cell lymphoma involving the mediastinum, CD20-positive with a Ki-67 proliferation index of 40%, and BCL2 and C-MYC negative. The slides were subsequently reviewed at a second pathology center, which reported PMBCL on the basis of an expanded immunohistochemical panel (CD20-positive, CD3-negative, CD10-negative, CD23-positive in scarce cells, CD30 weakly positive, BCL6-positive, BCL2-positive, MUM1-positive, and MYC-positive in 20% of cells, with a Ki-67 proliferation index of 90%), and a morphology of large lymphoid cells with scant eosinophilic cytoplasm, round nuclei, open chromatin, and prominent nucleoli, arranged in a diffuse pattern with compartmentalizing fibrous bands. Hematology classified the disease clinically as Ann Arbor stage IV [6], based on the pleural and pericardial effusions, without additional staging by PET-CT or bone marrow biopsy, which were not performed.

Chemotherapy with R-CHOEP (rituximab, cyclophosphamide, doxorubicin, vincristine, etoposide, and prednisone) was initiated shortly after diagnosis. One week later, he was readmitted with febrile neutropenia, complicated by concurrent SARS-CoV-2 infection and a new bilateral pleural effusion requiring drainage of the left side; no causative organism was isolated, and he improved with supportive management, without recurrence of pericardial effusion or hemodynamic compromise. Longer-term oncologic follow-up is beyond the scope of this report, which focuses on the index presentation, diagnostic evaluation, and acute management of tamponade.

## Discussion

Pericardial effusion severe enough to cause tamponade demands simultaneous hemodynamic stabilization and etiologic investigation, consistent with the structured diagnostic algorithm recommended by contemporary guidelines on pericardial disease [7]. In this patient, three features suggested a neoplastic cause before any tissue was obtained: a three-week constitutional syndrome of diaphoresis, fever, and weight loss; the serosanguineous character of the fluid; and an exudative biochemical profile with markedly elevated lactate dehydrogenase.

The second, and more broadly transferable, contribution of this case concerns the setting in which stabilization occurred. The patient presented to a rural hospital without a cardiologist or cardiothoracic surgeon on site, where the conventional options would have been transfer of an unstable patient to a distant referral center or an emergency procedure without specialist support. Instead, real-time telemedicine supervision allowed an internist to perform pericardiocentesis successfully before referral, with no immediate procedural complications, consistent with evidence that telehealth consultation in rural emergency departments improves access to specialist opinion, procedural support, and transfer decisions without evidence of harm [8,9]. Real-time remote guidance of the procedure itself, telementoring, has a more specific evidence base in point-of-care ultrasonography, where it improves diagnostic accuracy and confidence in inexperienced operators, though most experience concerns image interpretation rather than an invasive procedure [10]. Extending telementoring to a genuinely invasive, time-critical procedure, as here, illustrates a less-described application: moment-to-moment guidance of the procedure itself. Because delay for interfacility transfer can itself be fatal in tamponade, a locally performed, remotely supervised procedure, rather than transfer before drainage, was the strategy most likely to preserve hemodynamic stability in this setting [7].

The elevated adenosine deaminase level of 200 IU/L deserves comment. Pericardial fluid adenosine deaminase has been reported to have high diagnostic value for tuberculous pericarditis, with widely cited threshold values in the range of 30-40 IU/L [11,12]. However, adenosine deaminase is not specific for mycobacterial infection: it is released by activated, proliferating T-lymphocytes and can also be substantially elevated in lymphoproliferative and other malignant effusions, producing a well-recognized biochemical overlap between tuberculous and malignant pericarditis [11]. In this patient, the acid-fast bacilli smear and mycobacterial culture were both negative, while imaging demonstrated a large necrotic anterior mediastinal mass, a recognized diagnostic pitfall that reinforces the principle that tissue diagnosis, not fluid biochemistry alone, resolves the etiology [11,12].

Malignant disease accounts for a substantial share of moderate-to-large pericardial effusions in referral practice, with lung and breast carcinoma the leading solid-tumor causes and lymphoma the most frequent hematologic cause [13]. Pericardial involvement in non-Hodgkin lymphoma can arise by direct extension from a bulky mediastinal mass, as in this patient, or by hematogenous or lymphatic seeding, either producing an exudative, often serosanguineous effusion that progresses to tamponade before a diagnosis is established; tamponade as the initial manifestation of lymphoma is reported considerably more often with Hodgkin lymphoma than PMBCL, which is what makes systematic reporting of cases like this one relevant to the differential of unexplained tamponade [4]. The anterior mediastinal location narrowed the differential to thymoma, germ cell tumor, and lymphoma; normal alpha-fetoprotein and beta-human chorionic gonadotropin argued against a germ cell tumor, and definitive classification required histopathology obtained by video-assisted thoracoscopic surgery, which combined diagnosis with definitive management through a pericardial window, avoiding the recurrence risk of simple drainage, since effusion frequently reaccumulates after needle pericardiocentesis alone when the visceral pericardium remains tumor-involved [13]. A surgically created pericardial window establishes a durable communication that allows continued drainage and carries a lower recurrence rate than pericardiocentesis alone, with the thoracoscopic approach additionally permitting direct visualization and biopsy under a single anesthetic, precisely what allowed simultaneous diagnosis and definitive management in this patient [14,15].

Once tissue diagnosis established a large B-cell lymphoma involving the mediastinum, its distinction from diffuse large B-cell lymphoma, not otherwise specified (DLBCL-NOS), proved diagnostically demanding. PMBCL is a molecularly distinct entity sharing gene expression and genetic features with classical Hodgkin lymphoma, including frequent 9p24.1 alterations encoding PD-L1/PD-L2, rather than fitting the germinal-center/non-germinal-center framework (e.g., the Hans algorithm) used to subclassify DLBCL-NOS [5,16]. In this patient, the initial biopsy read (CD20-positive, Ki-67 40%, BCL2 and C-MYC negative) was compatible with, but not specific to, either entity; review at a second pathology center subsequently supported PMBCL based on an expanded immunophenotype (CD23 positivity in scarce cells, weak CD30 positivity, and BCL6/MUM1 positivity), and, notably, compartmentalizing sclerosis on morphology, a recognized hallmark of PMBCL. That the two readings disagreed on BCL2, C-MYC, and the Ki-67 index illustrates a recurring diagnostic challenge with mediastinal large B-cell lymphomas: overlapping morphology and immunophenotype with DLBCL-NOS mean classification can be sensitive to panel composition and interobserver interpretation, and dedicated hematopathology review is often needed to resolve it [5]. A validated prognostic index, the International Prognostic Index or the NCCN-IPI, remains useful at diagnosis even though most PMBCL presents as localized, low-risk disease; this patient's bulky disease, pleural and pericardial effusion, clinical stage IV, and markedly elevated lactate dehydrogenase instead portend a more advanced, higher-risk presentation [17].

This report has several limitations: it describes a single patient, so conclusions about generalizability cannot be drawn; the telemedicine-guided approach to pericardiocentesis was enabled by an ad hoc collaboration between a rural internist and a remote cardiologist rather than a structured institutional protocol, and its safety and reproducibility would need systematic evaluation before broader adoption; the two pathology readings of the mediastinal biopsy disagreed on BCL2, C-MYC, and the Ki-67 proliferation index, reflecting a recognized source of diagnostic uncertainty in mediastinal large B-cell lymphomas; and bone marrow biopsy, PET-CT, and formal calculation of a prognostic index were not completed during the index hospitalization, with staging relying on clinical assessment alone. Finally, this report focuses deliberately on the index presentation and the initiation of chemotherapy; longer-term oncologic follow-up is intentionally beyond its scope.

## Conclusions

Cardiac tamponade is an uncommon but recognized initial presentation of non-Hodgkin lymphoma, including PMBCL, as in this patient. Malignancy should be considered early in any large pericardial effusion accompanied by constitutional symptoms, serosanguineous fluid, and an exudative profile with high lactate dehydrogenase, even when adenosine deaminase might otherwise suggest tuberculosis. Fluid analysis narrows the differential but does not replace tissue diagnosis, and a combined approach such as video-assisted thoracoscopic surgery with pericardial window and biopsy is well suited to this scenario. Finally, in this patient, real-time telemedicine guidance allowed an internist to complete an emergent bedside pericardiocentesis without immediate complications; whether this model can be applied more broadly requires systematic evaluation.
